# The performance of AI in medical examinations: an exploration of ChatGPT in ultrasound medical education

**DOI:** 10.3389/fmed.2024.1472006

**Published:** 2024-11-05

**Authors:** Dao-Rong Hong, Chun-Yan Huang

**Affiliations:** ^1^Department of Ultrasonography, The Second Affiliated Hospital of Fujian Medical University, Quanzhou, Fujian, China; ^2^Department of General Practice, The Second Affiliated Hospital of Fujian Medical University, Quanzhou, Fujian, China

**Keywords:** ChatGPT, ultrasound medicine, medical education, artificial intelligence (AI), examination

## Abstract

**Objective:**

This study aims to evaluate the accuracy of ChatGPT in the context of China’s Intermediate Professional Technical Qualification Examination for Ultrasound Medicine, exploring its potential role in ultrasound medical education.

**Methods:**

A total of 100 questions, comprising 70 single-choice and 30 multiple-choice questions, were selected from the examination’s question bank. These questions were categorized into four groups: basic knowledge, relevant clinical knowledge, professional knowledge, and professional practice. ChatGPT versions 3.5 and 4.0 were tested, and accuracy was measured based on the proportion of correct answers for each version.

**Results:**

ChatGPT 3.5 achieved an accuracy of 35.7% for single-choice and 30.0% for multiple-choice questions, while version 4.0 improved to 61.4 and 50.0%, respectively. Both versions performed better in basic knowledge questions but showed limitations in professional practice-related questions. Version 4.0 demonstrated significant improvements across all categories compared to version 3.5, but it still underperformed when compared to resident doctors in certain areas.

**Conclusion:**

While ChatGPT did not meet the passing criteria for the Intermediate Professional Technical Qualification Examination in Ultrasound Medicine, its strong performance in basic medical knowledge suggests potential as a supplementary tool in medical education. However, its limitations in addressing professional practice tasks need to be addressed.

## Background

The third digital revolution, driven by rapid AI advancements, has brought about transformational change. A key development is the rise of chat models like ChatGPT, marking a major milestone in AI. Chat Generative Pre-trained Transformer (ChatGPT), as a disruptive technology, extends its influence globally, especially in medical education (1–5).

Although ChatGPT was not specifically trained for medical purposes, it has shown potential in applications such as medical data summarization, writing, and education (6). Recent advancements have seen an escalation in the integration of ChatGPT within medical disciplines. Gilson et al. (7) conducted a comparative study and established that ChatGPT’s proficiency in medical knowledge assessment rivals that of third-year medical students in the United States, underscoring its potential utility in medical education. Further, Antaki et al. (8) appraised its application in ophthalmology, discovering its performance paralleled the competence of first-year resident doctors, thereby highlighting ChatGPT’s exceptional capabilities and bolstering its perceived efficacy. Notably, even without bespoke training in radiology, ChatGPT has demonstrated remarkable aptitude in radiological evaluations (9). Complementing these findings, Sabry et al. (10) corroborated that ChatGPT, devoid of specialized medical training, nonetheless exhibits potential in providing clinical diagnostic assistance. Collectively, these empirical investigations illuminate the expansive applications of ChatGPT across diverse medical specialties.

Despite these advancements, the application of ChatGPT in ultrasound medicine remains nascent. This study delves into an evaluative analysis of ChatGPT’s effectiveness in the context of the Chinese Intermediate Professional Technical Qualification Examination for Ultrasound Medicine. The objective is to discern its prospective educational implications within this specialized medical field.

## Methods

In this empirical study, a total of 100 questions, comprising 70 single-choice and 30 multiple-choice items, were randomly selected from the question bank designated for the Chinese Intermediate Professional Technical Qualification Examination in Ultrasound Medicine, ensuring a balanced representation of the exam content. This examination comprehensively covers four distinct domains: basic knowledge, relevant clinical knowledge, professional knowledge, and professional practice, with each domain represented by 25 questions.

**Figure 1:**
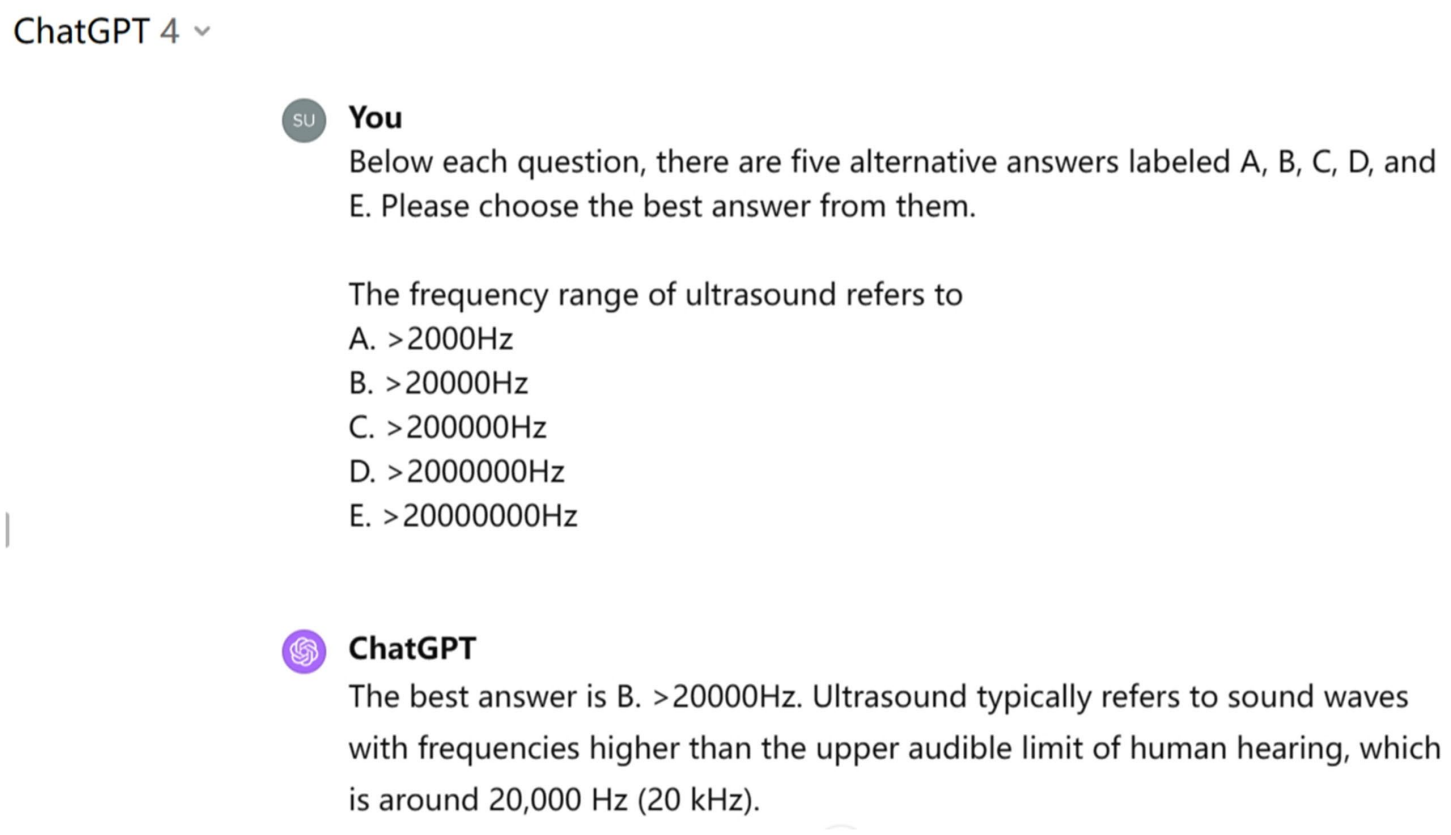
The questions were inputted and presented to ChatGPT for response.

For the purpose of this evaluation, these questions were systematically inputted and presented to both ChatGPT 3.5 and 4.0 for response, as shown in Figure 1. In parallel, a cohort of resident doctors, representing the human professional standard, was also engaged to answer the identical set of questions. Responses obtained from both the AI models and the resident doctors were meticulously recorded.

The primary outcome measure, accuracy rate, was determined through a comparative analysis of the responses against the correct answers, as stipulated in the examination’s answer key. This metric was calculated as the proportion of correct responses out of the total responses given.

This investigative exercise was executed over a span of 5 days, from March 5th to 10th, 2024, utilizing the aforementioned versions of ChatGPT. It is noteworthy that the Chinese Intermediate Professional Technical Qualification Examination in Ultrasound Medicine is a standardized national assessment, integral to the career progression framework for ultrasound medical professionals in China, aimed at evaluating their comprehensive knowledge and skills in the field.

### Statistics

To ascertain the accuracy, the responses from ChatGPT and resident doctors were meticulously compared against the correct answers in the question bank. Data analysis was conducted using Microsoft Excel, enabling the calculation and presentation of results as percentages.

## Results

In this assessment, ChatGPT 3.5 achieved a total score of 34 points, registering an accuracy of 35.7% in single-choice and 30.0% in multiple-choice questions. Detailed accuracy rates revealed 52.0% in basic knowledge, 44.0% in relevant clinical knowledge, 24.0% in professional knowledge, and a modest 16.0% in professional practice. In contrast, ChatGPT4.0 garnered 58 points, exhibiting enhanced accuracy rates of 61.4 and 50.0% in single-choice and multiple-choice questions, respectively. This version achieved notably higher accuracies: 76.0% in basic knowledge, 68.0% in relevant clinical knowledge, 48.0% in professional knowledge, and 40.0% in professional practice.

**Figure 2:**
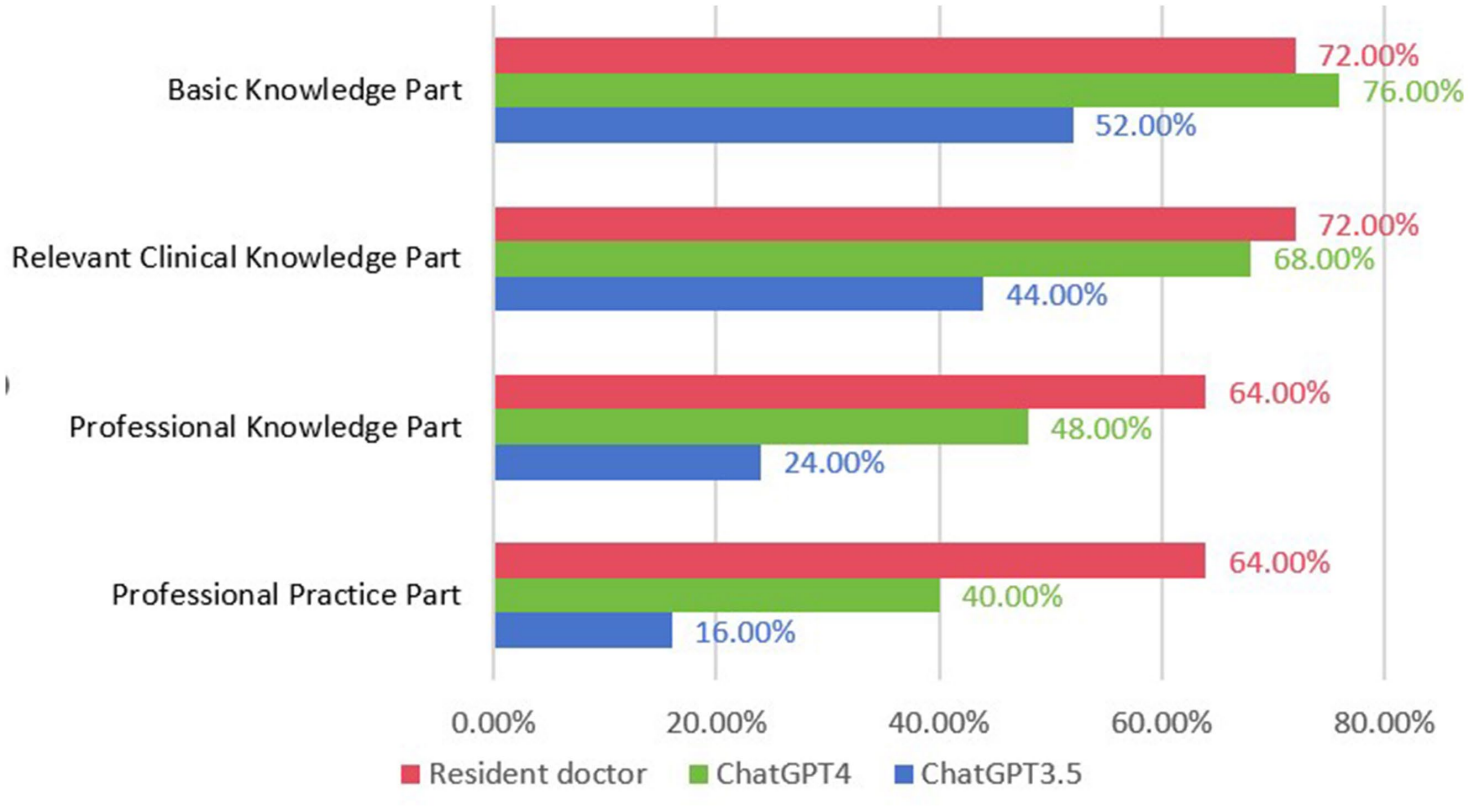
Compare the accuracy of ChatGPT3.5, ChatGPT4, and residents in each part of the questions.

Resident doctors, representing the human benchmark, scored 68 points. They demonstrated superior accuracy rates of 72.8% in single-choice and 53.3% in multiple-choice questions. Their detailed performance included 72.0% in both basic and relevant clinical knowledge, 64.0% in professional knowledge, and 64.0% in professional practice, as shown in Figure 2.

Notably, ChatGPT exhibited better results in single-choice questions as compared to multiple-choice ones. Version 4.0 showed a marked improvement in accuracy across all categories when compared to version 3.5. Both AI versions achieved their highest accuracies in basic knowledge, outperforming in relevant clinical knowledge, professional knowledge, and professional practice. However, resident doctors outshined both versions, particularly in professional knowledge and practice, while achieving comparable results to ChatGPT 4.0 in basic and relevant clinical knowledge.

## Discussion

ChatGPT, an AI chatbot created by OpenAI, carries substantial societal implications and has already been utilized in numerous scientific and medical applications (11, 12). The innovative use of conversational AI models like ChatGPT offers a potential advantage in delivering more accurate and timely medical information. While ChatGPT has shown notable advancements, its precision in specific medical subfields remains a topic of ongoing research (13).

This study illustrates that despite not achieving the passing criteria in China’s Intermediate Professional Technical Qualification Examination for Ultrasound Medicine, ChatGPT, especially its 4.0 version, exhibited considerable accuracy in basic and relevant clinical knowledge. These results are indicative of the progressive sophistication inherent in AI models through iterative development. As shown in Table 1, we summarize the advantages, disadvantages and future applications of AI in ultrasound medicine education. ChatGPT’s capabilities in various medical specialty exams highlight its utility in medical question-answering. Previous studies, such as by Gilson et al. (7), have demonstrated ChatGPT’s ability to match the average scores of third-year medical students in the United States, showcasing its potential as an interactive educational tool. Additionally, findings by Zhu et al. (14) and Tsang (15) suggest its effectiveness in clinical knowledge assessment and potential applications in undergraduate medical education, aligning with our observations of ChatGPT’s usefulness, particularly in the realm of ultrasound medicine exams. ChatGPT’s performance in specialized fields like ultrasound medicine could improve by fine-tuning it with specific medical datasets and adding more practical clinical scenarios to its training. This would help the model better manage complex diagnostic and operational tasks, addressing its current limitations in professional practice.

**Table 1:** AI in ultrasound medicine teaching: advantages, disadvantages, and future applications.

Aspect	Advantages	Disadvantages	Future exploration and applications
Education efficiency	AI models show high accuracy in basic and clinical knowledge.	Poor performance in professional practice, unable to compete with resident doctors.	Research how to combine AI training with specific medical scenarios to improve performance in professional practice.
Personalized learning	AI can adjust learning content to student needs, especially effective in basic medical knowledge.	Requires extensive data input and high maintenance costs.	Develop lower-cost, lower-data-demand personalized learning systems.
Assessment and feedback	Provides real-time feedback, helping students understand their learning status timely.	Potential biases in assessments, especially on complex medical issues.	Improve AI algorithms to reduce bias and increase assessment accuracy.
Technology application	Good grasp of basic medical knowledge and some clinical knowledge through AI.	Underperforms in handling complex clinical operations and judgments.	Develop more specialized AI models for specific needs in ultrasound medicine.
Remote teaching	AI enhances the efficiency of remote education, especially in the transfer of basic knowledge.	High dependence on technology may affect teaching quality due to technical issues.	Improve the stability and accessibility of remote teaching platforms.
Future applications	Advancements in AI could support broader medical training and professional development in the future.	Current AI models lack deep understanding of complex medical scenarios.	Use real clinical cases and operational data to train AI, enhancing its clinical decision-making capabilities.

However, when it comes to in-depth professional knowledge analysis, ChatGPT’s performance is somewhat inferior to that of human professionals. Both the 3.5 and 4.0 of ChatGPT performed best in basic knowledge domains but showed limitations in professional practice scenarios. ChatGPT exhibited comparable performance in a Master of Business Administration exam, excelling in basic questions but struggling with more complex process analysis tasks. This may reflect the current boundaries of AI in dealing with complex, practice-intensive tasks. While ChatGPT shows promise in analyzing basic ultrasound issues and techniques, its proficiency in handling intricate operational details requires further enhancement through advanced learning techniques. To address these limitations, future development could include real-world clinical scenarios and operational data in the model’s training. This would help the AI better understand and replicate the decision-making needed in complex clinical settings.

The findings highlight both the potential and limitations of AI in medical education. ChatGPT’s strengths in basic knowledge questions position it as a valuable auxiliary tool for medical education, reinforcing theoretical understanding. Nonetheless, its relative inadequacy in professional practice areas underlines that AI is not yet ready to fully supplant traditional clinical education, particularly in disciplines demanding in-depth clinical judgment and operational acumen.

## Conclusion

This research shows that while ChatGPT did not pass China’s Intermediate Professional Technical Qualification Examination in Ultrasound Medicine, it demonstrated strong basic medical knowledge and potential for medical education. With improvements like targeted training and clinical data integration, its performance could improve.AI models like ChatGPT can support traditional medical education by offering personalized learning and better access to training. As AI advances, it may play a bigger role in medical training and professional development.

Future studies should focus on creating specialized models, using real clinical cases, and exploring AI’s role in continuous medical education to enhance its effectiveness in fields like ultrasound medicine.

## Data Availability

The original contributions presented in the study are included in the article/supplementary material, further inquiries can be directed to the corresponding author.
